# Observer variability in the assessment of renal ^18^F-FDG uptake in kidney transplant recipients

**DOI:** 10.1038/s41598-020-61032-z

**Published:** 2020-03-12

**Authors:** Alexandre Jadoul, Pierre Lovinfosse, Antoine Bouquegneau, Laurent Weekers, Hans Pottel, Roland Hustinx, François Jouret

**Affiliations:** 1 Division of Nuclear Medicine and oncological imaging, University Hospital of Liege, Liege, Belgium; 2 Division of Nephrology, Department of Internal Medicine, University Hospital of Liege, Liege, Belgium; 3 Department of Public Health and Primary Care, KU Leuven Campus Kulak Kortrijk, Kortrijk, Belgium; 4 Groupe Interdisciplinaire de Géno-protéomique Appliquée, Cardiovascular Sciences, University of Liège, Liège, Belgium

**Keywords:** Medical research, Nephrology

## Abstract

^18^F-FDG PET/CT imaging may help non-invasively disprove the diagnosis of acute kidney allograft rejection (AR) in kidney transplant recipients (KTR). The present study aims at evaluating the repeatability and reproducibility of the quantification of renal ^18^F-FDG uptake in KTR. We prospectively performed ^18^F-FDG PET/CT in 95 adult KTR who underwent surveillance transplant biopsy between 3 to 6 months *post* transplantation. Images were obtained 180 minutes after injecting 3 MBq ^18^F-FDG per kg body weight. Mean standard uptake value (SUV_mean_) of kidney cortex was independently measured by 2 experienced observers in 4 volumes of interest (VOI) distributed in the upper (n = 2) and lower (n = 2) poles. The first observer repeated SUV assessment in the uppermost VOI, blinded to the initial results. Intra-class correlation coefficients (ICC) and Bland-Altman plots were calculated. An ICC of 0.96 with 95%CI of [0.94; 0.97] was calculated for the intra-observer measurements. The ICC for inter-observer reproducibility for each VOI was 0.87 [0.81–0.91], 0.87 [0.81–0.91], 0.85 [0.78–0.89] and 0.83 [0.76–0.88] for the upper to the lower renal poles, respectively. The repeatability and reproducibility of the quantification of kidney allograft ^18^F-FDG uptake are both consistent, which makes it transferrable to the clinical routine.

## Introduction

Kidney transplantation represents the treatment of choice for patients with end stage renal disease^1^. Despite the steady progress of immunosuppressive treatments, acute rejection (AR) remains a recurrent complication which impacts both graft and patient survivals^2,3^. Furthermore, systematic studies focusing on the clinical value of protocol biopsies (by definition performed in stable kidney transplant recipients (KTR)) have demonstrated a non-negligible prevalence of subclinical AR^4–8^. By definition, subclinical AR corresponds to “the histological documentation of unexpected evidence of AR in a stable patient”^9^. Early management of AR decreases the risk of chronic cellular/humoral rejection, late AR episodes and improves long-term graft survival in KTR^10^. Therefore, precocious detection of (subclinical) AR is essential.

In current clinical practice, transplant needle biopsy (TNB) using Banff classification is the gold standard for AR diagnosis^11^. Still, it is associated with a substantial risk of complications, such as hemorrhage or infection^12^. Thus, non-invasive approaches have been developed over the past decades in order to help clinicians avoid potential side effects of TNB^13–15^. Particularly, promising preclinical and clinical observations have been reported on the role of ^18^F-fluorodeoxyglucose (^18^F-FDG) positron-emission tomography coupled with computed tomography (PET/CT) in kidney allograft AR, in both diagnosis and therapeutic monitoring^16–18^. One may speculate that the AR-induced recruitment of activated leukocytes – with high metabolic activity – increases the uptake of ^18^F-FDG in renal graft cortex^19^.

In addition to the visual assessment, PET/CT allows a semi-quantitative analysis of the images which is reflected by standardized uptake value (SUV). SUV represents the decay-corrected concentration of intravenously injected ^18^F-FDG in a volume of interest (VOI). Doing so, we demonstrated a significant link between cortical renal graft SUV_mean_ and Banff score, with sensitivity and specificity in diagnosing AR of 100% and 50%, respectively, using a threshold of 1.6^16^. However, many well-known factors may affect the accuracy of SUV measurement, including patient weight, blood glucose level, time between the injection of the ^18^F-FDG and image acquisition, partial-volume effect, and recovery coefficient. Additionally, VOI delineation, which is the sole parameter dependent on the physician, may bias the quantification of renal ^18^F-FDG uptake^20^. The segmentation of kidney transplant is especially important in order to avoid VOI contamination by the physiological activity linked to the urine excretion of the radiotracer. The purpose of this study is therefore to evaluate the intra- and inter-observer variability in the assessment of renal ^18^F-FDG uptake in KTR.

## Material and Methods

### Patients

From November 2015 to January 2018, we prospectively performed an ^18^F-FDG PET/CT imaging in KTR undergoing a surveillance transplant biopsy between 3 to 6 months post transplantation. Patients with delayed protocol biopsy (>6 months), under 18 years, who underwent transplantectomy, or who were pregnant or breastfeeding were all excluded. Estimated glomerular filtration rate (eGFR) was calculated using modification of diet in renal disease (MDRD) equation. The study was approved by the institutional review board of the University of Liege.

### ^18^F-FDG PET/CT

PET/CT was performed using cross-calibrated Philips GEMINI TF Big Bore or TF 16 PET/CT systems (Philips Medical Systems, Cleveland, OH). Low-dose helical CT (5-mm slice thickness, 120-kV tube voltage, and 40-mAs tube current–time product) was followed by a PET emission scanning with 2 bed positions each lasting 240 seconds. Image reconstruction involved iterative list mode time-of-flight algorithms. Corrections for attenuation, dead time, random, and scatter events were applied.

Mean standard uptake value (SUV_mean_) of kidney cortex was measured by 2 observers (board-certified physicians in nuclear medicine with 9 and 5 year-experience in ^18^F-FDG PET/CT imaging) in 4 VOI of 1 mL distributed in the upper (n = 2) and lower (n = 2) poles at distance of the pelvicalyceal zone. There was no *a priori* minimal threshold of distance to draw the VOI from the urinary pelvis. One VOI of 20 mL was drawn in the psoas muscle. The observer 1 repeated SUV assessment in the uppermost VOI, blinded to the initial results. SUV_mean_ of each VOI was calculated with the following formula: [Voxel Value (Bq/mL) × Patient Weight (kg)]/[Injected Dose (Bq) × 1000 (g/kg)]. On average, it takes ~5 minutes to measure the SUV_mean_ of the renal cortex and the psoas muscle per patient.

### Statistics

To measure the agreement between the results (intra- and inter-observer variability), the following statistical methods were used: Repeated measures ANOVA, Bland and Altman’s graph, and intra-class correlation coefficient (ICC). ICC is a measure of the agreement between two methods when the studied variable is continuous. Closer the ICC is to 1, better is the agreement between the two measurements. The results are considered significant at the significance level of 5% (p < 0.05).

### Ethical approval and consent to participate

All procedures were performed in accordance with the principles of the 1964 Declaration of Helsinki and its later amendments or comparable ethical standards. The study design and exemption from informed consent were approved by the Institutional Review Board of Liege University Hospital.

## Results

Ninety-five adult KTR underwent one PET/CT between November 2015 and January 2018, within 3 to 6 months following the transplantation. The mean age of the cohort was 54 ± 13 years (Range: 19–73 y.), with a male to female ratio of 2.4. Characteristics of the cohort are summarized in Table 1. PET/CT was performed in fasting conditions 195 ± 14 (Range: 174–231) min after injection of 245 ± 32 (Range: 156–350) MBq of ^18^F-FDG. Mean glycemia was 109 ± 27 (Range: 62–241) mg/dL.

**Table 1:** Characteristics of the population.

		Cohort (n = 95)
Recipients	Sex [M/F (n); sex ratio]	67/28; 2.4
	BMI (Kg/m2) mean ± stand dev	26 ± 4
	PRA max (n) [<5%/5%–85%/>85%]	86/6/3
Donors	Sex [M/F (n); sex ratio]	57/38; 1.5
	Donor type (n) [DBD/DCD/LD]	69/20/6
	BMI (Kg/m^2^) mean ± stand dev	25 ± 5
Transplantation	Rank (n)[1st/2nd/3rd]	84/9/2
	CIT (min) mean ± stand dev	699 ± 289
	HLA mm mean ± stand dev	
	A	0.89 ± 0.69
	B	1.21 ± 0.64
	DR	0.73 ± 0.57
	Early graft function (n) [immediate/slow/delayed]	67/19/9
Status at the time of biopsy	Maintenance immunosuppression (n)	
	-CNI [CsA/FK/none]	
	-Antimetabolite	2/93/0
	[MMF/MPA/AZA/none]	
	-mTOR inhibitor [yes/no]	82/7/0/6
	-Steroids [yes/no]	1/94
		93/2
	Number of days between KTx and biopsy (mean ± SD)	105 ± 27
	Creatinine (mg/dL) mean ±SD	1.41 ± 0.44
Type of rejection	None/Borderline/Cellular Rejection	73/16/6

Abbreviations: AZA, azathioprine; BMI, body mass index; CNI, calcineurin inhibitors; CS, Corticosteroids; CsA, cyclosporin A; DCD, donor after circulatory death; DBD, donor after brain death; FK, tacrolimus; KTx, kidney transplantation; LD, living donor; MMF, mycophenolate mofetyl; MPA, mycophenolic acid; mTOR, mammalian target of rapamycin.

**Figure 1:**
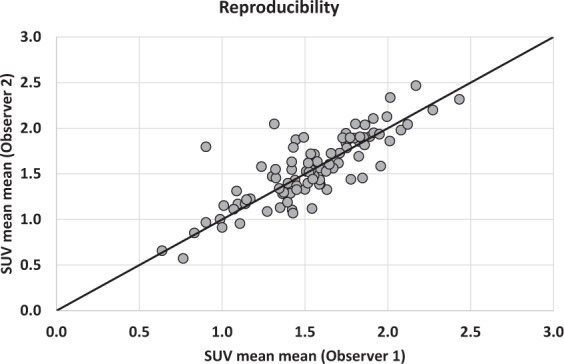
Correlation of mean of SUV_mean_ of the 4 VOI between observer 1 and observer 2. The line depicts the perfect concordance.

**Figure 2:**
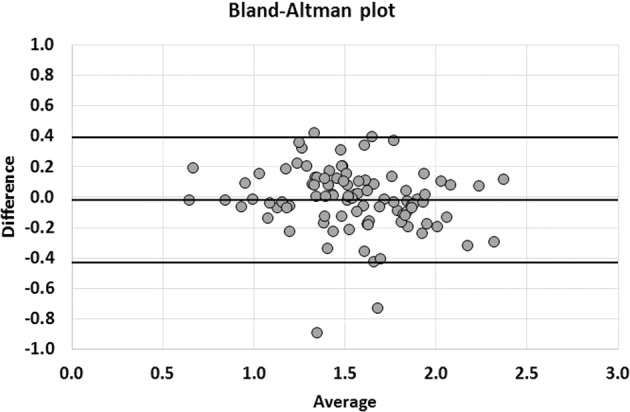
Bland-Altman graph of mean of SUV_mean_ of the 4 VOI. The middle line represents the average of the differences between observer 1 and observer 2 and the two other lines the mean of the differences ± 1.96 standard deviations.

**Figure 3:**
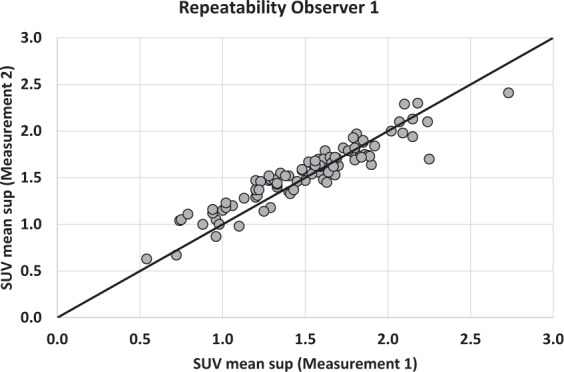
Correlation of SUV_mean_ of the uppermost VOI between measurement 1 and measurement 2 by the same physician. The line depicts the perfect concordance.

**Figure 4:**
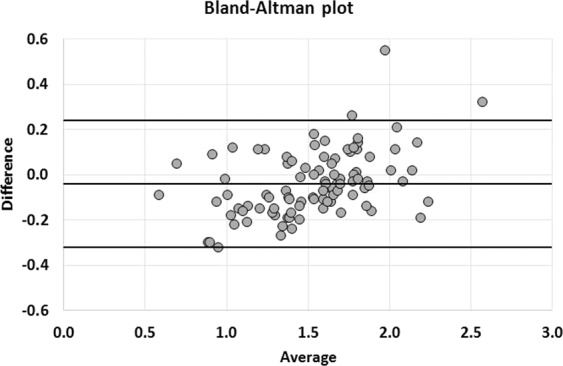
Bland-Altman graph of SUV_mean_ of the uppermost VOI. The middle line represents the average of the differences between measurement 1 and measurement 2 by the same physician and the two other lines the mean of the differences ± 1.96 standard deviations.

The values of kidney transplant SUV_mean_ were, from top to bottom, 1.51 ± 0.40 (Range: 0.54–2.73), 1.54 ± 0.36 (Range: 0.6–2.44), 1.55 ± 0.36 (Range: 0.65–2.57), 1.56 ± 0.37 (Range: 0.64–2.68) in observer 1 and 1.53 ± 0.37 (Range: 0.51–2.53), 1.55 ± 0.38 (Range: 0.57–2.44), 1.58 ± 0.39 (Range: 0.65–2.53), 1.58 ± 0.38 (Range: 0.56–2.5) in observer 2. No significant difference was observed between the SUV_mean_ of the 4 VOIs of the same kidney (p = 0.41) in observer 1, while SUV_mean_ of the superior pole was significantly lower than the 3 others values in observer 2 (p = 0.0001). There was a strong correlation between the 2 observers with ICC values of 0.87, 0.87, 0.85 and 0.83, respectively for each VOI. A slightly better correlation was observed when considering the mean of the 4 values (ICC: 0.90) (Fig. 1), but with no significant difference compared to correlation for each pole (p-value = 0.29, 0.26, 0.11, 0.06). Bland Altman analyses showed mean differences of −0.01 [−0.07; +0.04], −0.01 [−0.06; +0.04] for the upper poles and −0.03 [−0.08; +0.03], −0.02 [−0.08; +0.04] for the lower poles. The difference for the mean of these values was −0.02 [−0.06; +0.03] (Fig. 2). Concerning the repeatability, the agreement was calculated for the intra-observer measurements (ICC: 0.96) (Fig. 3), with mean difference of −0.04 [−0.07; −0.01] in Bland Altman graph (Fig. 4). The same statistics were performed for SUV_max_ and showed lower values both for ICC and Bland Altman analyses (Annex 1).

**Figure 5:**
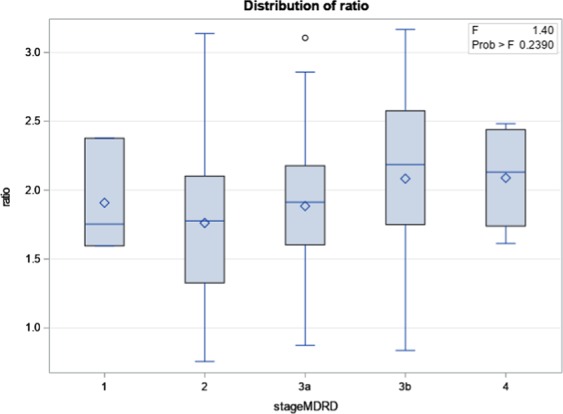
Correlation between estimated eGFR calculated by MDRD equation and ratio of mean kidney SUV_mean_/SUV_mean_ psoas. (n stage 1 = 3, n stage 2 = 25, n stage 3a = 35, n stage 3b = 28, n stage 4 = 4).

Finally, no significant relationship was highlighted between MDRD-based stages of chronic kidney disease (CKD) and the value of the ratio between mean kidney SUV_mean_ and SUV_mean_ of the psoas muscle (p = 0.24) (Fig. 5).

## Discussion

^18^F-FDG PET/CT functional imaging is a promising tool in the assessment of AR-associated inflammation^16,21,22^. This rapid imaging technique does not cause any side-effect in patients with renal failure from normal to mildly reduced GFR to end-stage renal disease. Furthermore, we have recently showed that ^18^F-FDG PET/CT may help rule out subclinical rejection in stable KTR, with a negative predictive value of 98%^23^. Our results support conclusions of previous studies which showed that the uptake of ^18^F-FDG by kidneys in non-transplant patients and in KTR is not influenced by renal function^24,25^, and that the alteration of GFR does not significantly compromise the clearance of background activity^26^. Moreover, the physiological urinary excretion of ^18^F-FDG may hamper the measurement of ^18^F-FDG uptake in the renal parenchyma^27^. To overcome this problem, we performed late acquisitions and drew multiple VOI. Although the SUV_max_ has shown the lower inter-observer variability in tumors^28,29^, we elected to use the SUV_mean_ in order to limit the impact of a potential urinary contamination in the VOI. Doing so, we observed a consistent agreement between the two observers for all VOI. The agreement was the best in the upper pole (ICC: 0.87). No previous data are available in the literature since ^18^F-FDG PET/CT imaging has been very recently tested in AR diagnosis in KTR^16^. However, Huang *et al*.^29^ and Büyükdereli *et al*.^30^ have demonstrated a high inter-observer correlation of SUV_mean_ in the evaluation of 43 pulmonary nodules (ICC: 0.97) and 97 lung lesions (ICC: 0.98), respectively. Benz *et al*.^28^ and Goh *et al*.^31^ also proved a strong reproducibility of SUV_mean_ assessment in treatment monitoring by ^18^F-FDG PET/CT in 33 patients with sarcoma (CCC: 0.84) and in the characterization of colorectal tumors (ICC: 0.85). The analysis of intra-observer variability has shown similar results with a high correlation between repeated measurements by the same physician^30,31^.

Despite the absence of significant difference between ICC of the mean of SUV_mean_ and SUV_mean_ of each pole, superior pole SUV_mean_ of observer 2 was significantly lower than the 3 other values. Furthermore, even if the delineation of only one single VOI is easier in clinical routine, we must keep in mind that histopathological changes in the kidney allograft may not be homogeneous. Sorof *et al*.^32^ highlighted discordances in histological grading in 30% and therapeutic defects in 7.5% of cases with only one analyzed sample. Automatic kidney component segmentation methods are currently under development and validation. Such an approach may help improve PET/CT-based diagnosis of kidney allograft rejection^33^. As a whole, we currently recommend to draw multiple VOI within the renal parenchyma rather than only one.

Although simplicity and ease of use are among the strengths of SUV, the measurement is nevertheless vulnerable to many sources of unwanted variability^20^. Despite a careful attention to protocol during the acquisition, there is still a within-subject coefficient of variation in SUV_mean_, reaching 10% for tumours (4.8 to 17.7 depending of the studies)^34^ and 10–15% for normal tissues^35^. This variability could be problematic for “borderline” cases with SUV right next to cutoff values.

## Conclusions

This study shows that assessment of renal ^18^F-FDG uptake in KTR is highly repeatable and reproducible if ^18^F-FDG PET/CT images are evaluated by experienced observers with careful attention to the technique.

## Supplementary information

Supplementary information.

## Data Availability

The datasets used and/or analysed during the current study are available from the corresponding author on reasonable request.
