# Supplementary material for: Observer variability in the assessment of renal 18F-FDG uptake in kidney transplant recipients
Source: Sci Rep. 2020 Mar 12;10:4617. doi: 10.1038/s41598-020-61032-z (PMC7067780; doi:10.1038/s41598-020-61032-z)
Supplement: Supplementary file 1 — Supplementary information. [file 41598_2020_61032_MOESM1_ESM.docx]

**Observer variability in the assessment of renal ^18^F-FDG uptake in kidney transplant recipients**

Alexandre Jadoul^1^, MD
Pierre Lovinfosse^1^, MD, PhD
Antoine Bouquegneau^2^, MD
Laurent Weekers^2^, MD
Hans Pottel^3^, PhD
Roland Hustinx^1^, MD, PhD
Francois Jouret^2, 4^, MD, PhD

*^1^Division of Nuclear Medicine and oncological imaging, University Hospital of Liege, Liege, Belgium
^2^Division of Nephrology, Department of Internal Medicine, University Hospital of Liege, Liege, Belgium
^3^Department of Public Health and Primary Care, KU Leuven Campus Kulak Kortrijk, Kortrijk, Belgium.
^4^Groupe Interdisciplinaire de Géno-protéomique Appliquée, Cardiovascular Sciences, University of Liège, Liège, Belgium*

**Supplementary data**

**Annex 1.**

ICC and mean differences between observer 1 and and observer 2 for SUVmax.

|  | Upper pole | Mean upper pole | Mean lower pole | Lower pole |
| --- | --- | --- | --- | --- |
| ICC | 0.85 | 0.86 | 0.81 | 0.81 |
| Mean diff (CI95%) | 0.04 [-0.04;+0.11] | -0.05 [-0.12+0.01] | -0.11 [-0.19; -0.04] | -0.08 [-0.15; 0.00] |
